# Assessment of Anatomical Uniqueness of Maxillary Sinuses through 3D–3D Superimposition: An Additional Help to Personal Identification

**DOI:** 10.3390/biology12071018

**Published:** 2023-07-18

**Authors:** Andrea Palamenghi, Annalisa Cappella, Michaela Cellina, Danilo De Angelis, Chiarella Sforza, Cristina Cattaneo, Daniele Gibelli

**Affiliations:** 1 LAFAS—Laboratorio di Anatomia Funzionale dell’Apparato Stomatognatico, Dipartimento di Scienze Biomediche per la Salute, Università degli Studi di Milano, Via Luigi Mangiagalli 31, 20133 Milano, Italy; 2 LABANOF—Laboratorio di Antropologia e Odontologia Forense, Dipartimento di Scienze Biomediche per la Salute, Università degli Studi di Milano, Via Luigi Mangiagalli 37, 20133 Milano, Italy; 3 Dipartimento di Scienze Biomediche per la Salute, Università degli Studi di Milano, Via Luigi Mangiagalli 31, 20133 Milano, Italy; 4 U.O. Laboratorio di Morfologia Umana Applicata, IRCCS Policlinico San Donato, 20097 San Donato Milanese, Italy; 5 Reparto di Radiologia, Ospedale Fatebenefratelli, ASST Fatebenefratelli Sacco, 20121 Milano, Italy

**Keywords:** personal identification, maxillary sinus, CT-scan, individual anatomy, 3D–3D superimposition

## Abstract

**Simple Summary:**

Personal identification is a paramount activity in forensic anthropology and can be achieved through the comparison of antemortem and postmortem images. Among the most individualizing skeletal structures are paranasal sinuses, which have been extensively investigated for identification purposes, with great emphasis on frontal sinuses. This paper extends this research line and assesses the uniqueness of the maxillary sinuses by applying 3D superimposition to investigate the reliability of these sinuses as reference anatomical structures for personal identification. Models of maxillary sinuses were acquired twice from CT scans to simulate antemortem and postmortem images; then, they were superimposed by pairing models from the same individual and from different individuals. The point-to-point distance between the models was used as a proxy to evaluate if the models belong to the same person. The optimal results suggest that maxillary sinus are reliable indicators of identity, although further research is needed to evaluate the performance of the method when the surface of the maxillary sinuses undergoes modifications due to pathological conditions.

**Abstract:**

Paranasal sinuses represent one of the most individualizing structures of the human body and some of them have been already analyzed for possible applications to personal identification, such as the frontal and sphenoid sinuses. This study explores the application of 3D–3D superimposition to maxillary sinuses in personal identification. One hundred head CT-scans of adult subjects (equally divided among males and females) were extracted from a hospital database. Maxillary sinuses were segmented twice from each subject through ITK-SNAP software and the correspondent 3D models were automatically superimposed to obtain 100 matches (when they belonged to the same person) and 100 mismatches (when they were extracted from different individuals), both from the right and left side. Average RMS (root mean square) point-to-point distance was then calculated for all the superimpositions; differences according to sex, side, and group (matches and mismatches) were assessed through three-way ANOVA test (*p* < 0.017). On average, RMS values were lower in matches (0.26 ± 0.19 mm in males, 0.24 ± 0.18 mm in females) than in mismatches (2.44 ± 0.87 mm in males, 2.20 ± 0.73 mm in females) with a significant difference (*p* < 0.001). No significant differences were found according to sex or side (*p* > 0.017). The study verified the potential of maxillary sinuses as reliable anatomical structures for personal identification in the forensic context.

## 1. Introduction

In forensic anthropology, personal identification is the ultimate step of the analysis of unknown human remains and consists of correctly assigning an identity to unknown decedents, based on the comparison between ante-mortem (AM) and post-mortem (PM) biological information [1]. The right to identity is enshrined in international laws and represents a compelling issue, especially in the wake of increasing migration flows and related mass disasters, with the consequent need for correctly identifying victims and returning their bodies to relatives [2].

Personal identification is most commonly performed through genetic, fingerprint and dental analyses [3,4]: however, anthropological methods may be reliably applied thanks to the individualizing potential of anatomical structures of the skeleton [5], including paranasal sinuses. Paranasal sinuses are air spaces excavated in cranial bones (frontal, maxillary, sphenoid and ethmoid bone): once they reach the final configuration, their shape is highly variable and unique for each individual, and are therefore the ideal structure to use for personal identification [6,7,8]. Traditionally, the morphological assessment of paranasal sinuses is performed through bidimensional comparisons between the silhouettes of sinuses in AM and PM material [7,9,10,11] or through the verification of correspondence in a series of morphological traits [12]. A novel and additional tool to easily compare the morphology of paranasal sinuses comes from the modern techniques of three-dimensional (3D) segmentation and 3D volume analysis. By extracting 3D models of paranasal sinuses from CTs and CBCT scans or NMR analyses, operators can superimpose anatomical structures obtained from AM and PM material [13,14]. This allows to quantify the surface similarity between 3D models of anatomical structures, hence obtaining a parameter to support a positive identification. Superimposition of 3D bone models has been applied also to post-cranial skeletal portions, suggesting the significant potential of this technique for personal identification [15] and other anthropological analyses [16,17,18,19,20].

So far, a few reports have verified the potential of 3D–3D superimposition of frontal and sphenoid sinuses for personal identification purposes [13,14,21]. As for maxillary sinuses, some radiographic studies suggested their potential as personal identifiers [22,23,24,25,26], whereas the 3D approach has not been tested yet. 

Maxillary sinuses are the largest paranasal sinuses and are excavated in the maxillary bone, lateral to nasal cavities and below orbits. They are limited superiorly by the orbital floor, medially by the lateral nasal wall, posteriorly by the anterior wall of the pterygopalatine fossa, inferiorly by the alveolar process of the maxillary bone, where the roots of posterior teeth may project into. Each maxillary sinus drains into the homolateral nasal cavity through the maxillary ostium in the middle meatus [27]. Maxillary sinuses begin to develop at 10 fetal weeks and increase in size until 20 years of age [28,29], and they are used also for sex assessment, being larger in males than in females [29,30,31,32,33,34]. Moreover, several works demonstrated that there is inter-population variability in the size and shape of maxillary sinuses, especially in relation to physiological functioning [35,36,37,38,39]. 

3D–3D superimposition procedures for the assessment of frontal and sphenoid sinuses have already demonstrated their great potential for personal identification; however, this approach has not yet been explored for maxillary sinuses. The present study aims at testing the 3D–3D superimposition procedure for personal identification purposes from the comparison of maxillary sinuses to verify the possible application as reference anatomical structures for AM–PM comparison in cases of personal identification of unknown decedents.

## 2. Materials and Methods

One hundred subjects (50 males and 50 females) who underwent head CT-scans were randomly chosen from a hospital database in Northern Italy. Exclusion criteria were craniofacial deformities, traumatic injuries involving the cranium, sinusitis, hypotrophy, or agenesia of maxillary sinuses. The age was 50 ± 20 years and 57 ± 24 years for males and females, respectively, without significant differences (Student’s *t*-test, *p* > 0.05). All CT scans were anonymized. The observational study was performed by virtue of the local and international ethical rules (approval by local ethical committee: 7331/2019). All CT scans were performed on a second-generation dual-source scanner, Somatom Definition Flash (Siemens, Forchheim, Germany), parameters of acquisition: kV 100, mAs 130, collimation 128 × 0.6 m, rotation time 1.0 s, reconstruction thickness 1 mm. 

**Figure 1:**
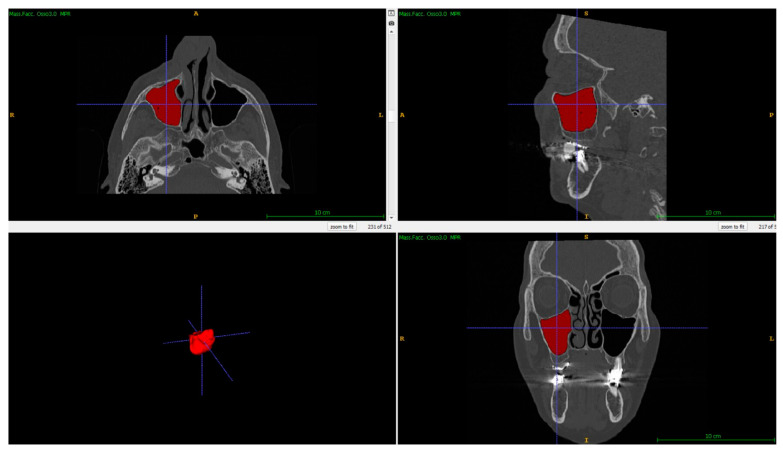
Segmentation of the right maxillary sinus through ITK-SNAP software: in the (**upper left**), (**upper right**) and (**lower right**) quadrants are the transversal, sagittal and coronary sections, respectively. In the (**lower left**) quadrant is the final 3D model of the maxillary sinus. The letters indicate the orientation of the anatomical structure.

The segmentation of maxillary sinuses from the 100 patients was performed twice through ITK-SNAP freeware via a semi-automatic procedure [6,17,40,41], which provides 3D models of areas with a specific gray range, involving only the air space of sinuses. Septa and other bone structure are not included in the model, so increasing the adherence of 3D with the individualizing characteristics of the maxillary sinuses. In detail, once both the right and left maxillary sinuses were selected, seeds that expanded according to the chosen gray level of acquisition were inserted into the air cavities, filling the space, and casting the volume of the sinus (Figure 1). 

**Figure 2:**
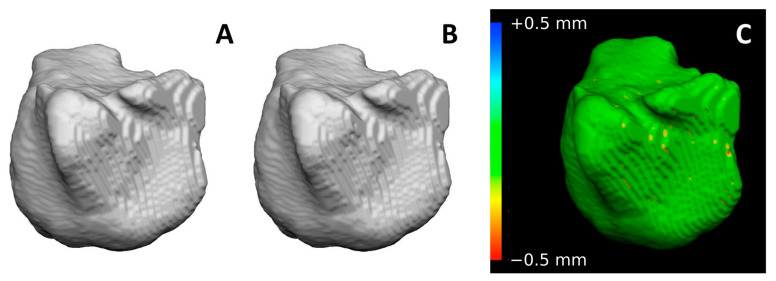
Superimposition of two maxillary sinuses segmented from CT scans belonging to the same individual: (**A**) AM model (first acquired model), (**B**) PM model (second acquired model), (**C**) chromatic visualization of surface differences between the two models according to RMS values: green color indicates that the two surfaces virtually correspond. On the left side, the legend indicates the point-to-point distance between the two models according to color map.

Repeatability of the semi-automatic segmentation has been already verified in a previous publication [42]. The first 3D segmentation of maxillary sinuses was considered the ante-mortem model (AM) in a fictitious identification, whereas the second 3D one was considered the post-mortem model (PM). The 3D models of the maxillary sinuses were then analyzed through VAM© software (version 2.8.3, Canfield Scientific Inc., Parsippany-Troy Hills, NJ, USA). The 3D models of right and left maxillary sinuses segmented from CT-scans of the same individual were then automatically superimposed according to the least point-to-point difference between the two surfaces, for a total of 200 superimpositions representing the group of matches (100 from the right side, 100 from the left side, equally divided among males and females, Figure 2). 

**Figure 3:**
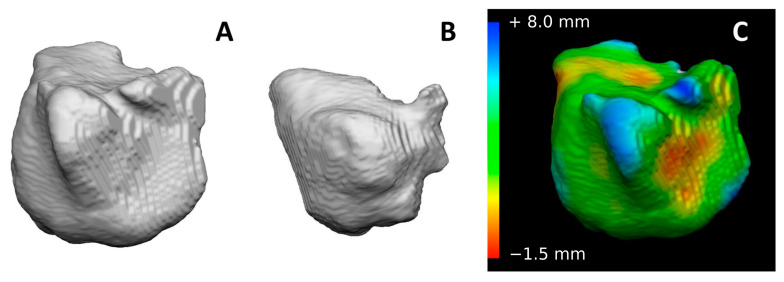
Superimposition of two maxillary sinuses segmented from CT scans belonging to different individuals: (**A**) AM model (first acquired model) of the first subject, (**B**) PM model (second acquired model) of the second subject, (**C**) chromatic visualization of surface differences between the two models according to RMS values: red, yellow and blue colors are predominant and indicate discrepancies between the two superimposed surfaces. On the left side, the legend indicates the point-to-point distance between the two models according to color map.

Secondarily, 3D models of right and left maxillary sinuses, extracted from CT-scans belonging to different individuals, were automatically superimposed according to the least point-to-point difference between the two surfaces, therefore producing 200 superimpositions representing the group of mismatches (100 from the right side, 100 from the left side, equally divided among males and females, Figure 3).

For each superimposition of matches and mismatches, the model classified as PM (second acquisition) was superimposed onto the model classified as AM (first acquisition). Once the registration between the two models was obtained both for matches and mismatches, VAM^®^ software was requested to calculate the RMS (root mean square) point-to-point distance, expressed in millimeters (mm). The RMS value is “the square root of the mean of the squared distances of each point of the model”, that is, output by VAM^®^ after the superimposition and registration [13,16,17]. The calculation of point-to-point distance between the two 3D models is accompanied by the chromatic visualization of surface differences, with areas colored in blue, green and red; blue and red colors correspond to areas more different between the models, whereas the green highlights unchanged areas. For all the RMS calculation, the AM model was chosen as reference [43].

Statistical analyses were performed using SPSS (IBM SPSS Statistics for Windows, Version 22.0, IBM Corp). Possible statistically significant differences in RMS values between matches and mismatches, males and females, and between the right and left sides were assessed through a three-way ANOVA *t*-test (*p* < 0.05), together with possible interactions among variables; Bonferroni correction was applied accordingly (*p* < 0.017).

## 3. Results

Mean RMS values in the superimposition of matches and mismatches are shown in Table 1. 

**Figure 4:**
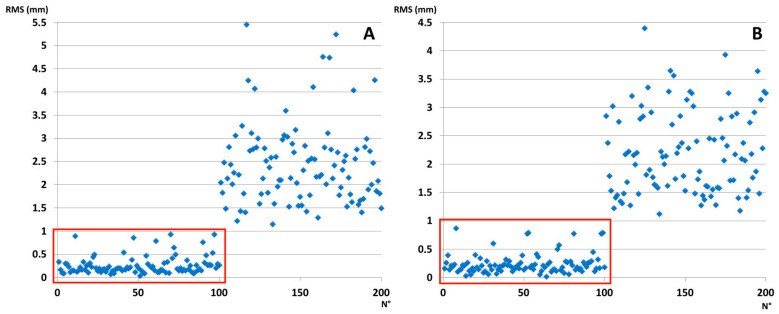
Dispersion of RMS in matches (0–100) and mismatches (101–200) in males (**A**) and females (**B**). Inside the red squares are the matches. The RMS value of 1 mm can efficiently distinguish matches from mismatches.

On average, the RMS value in matches was 0.26 ± 0.19 mm in males and 0.24 ± 0.18 mm in females, whereas in mismatches it was 2.44 ± 0.87 mm in males and 2.20 ± 0.73 mm in females, respectively. Matches always ranged between 0.02 mm and 0.93 mm, whereas mismatches were between 1.12 mm and 5.24 mm, without any overlap between the two groups (Figure 4). Generally, an arbitrary threshold of 1 mm can reliably distinguish matches and mismatches. 

Table 2 reports the results of the three-way ANOVA test: no statistically significant differences were found according to sex or side, and for any possible interactions (*p* > 0.017). However, a significant difference in RMS values was found between matches and mismatches, being lower in the former than in the latter group (*p* < 0.001) with a large effect size according to Cohen [44] (eta squared: 0.76).

## 4. Discussion

Personal identification represents one of the most sensitive procedures in forensic anthropology and is based on the comparison between AM biological data of an identity suspect and a similar set of information extracted from an unknown cadaver. This type of procedure is greatly helped by the natural individualizing potential of the osteological systems, as every anatomical structure can be used if adequate AM and PM material is available.

Among others, paranasal sinuses are considered the most variable structures in our body: the potential of the comparison of frontal sinuses silhouette for personal identification has been widely reported by the literature, thanks to their high visibility in conventional skull X-rays [12]. However, the increasing use of CT scans will enable anthropologists to easily assess other paranasal sinuses, with the additional improvement of the use of 3D segmentation procedures [40]. The chance of extracting 3D models of anatomical structures allows anthropologists to compare the entire surface of paranasal sinuses instead of their 2D silhouette. Since low intra/interobserver error was reported [17,42], the segmentation procedure can be easily repeated and reproduced. In addition, the 3D–3D superimposition outputs values that quantifies differences between 3D volumes adds a fundamental objective parameter of identification, which usually cannot be obtained through traditional bidimensional comparison methods. Therefore, although the procedure may take additional time, it provides sound and repeatable values that may support an identification [13,14,15,21,43].

So far, 3D–3D comparison methods have not been applied to maxillary sinuses: the present study aimed at filling this gap and verifying that these structures may be reliably used for personal identification. In fact, the groups of matches and mismatches do not show any overlap, with an arbitrarily chosen RMS threshold of 1.00 mm potentially useful to distinguish the two groups in 100% of cases. Interestingly, the results of this study are consistent with those from the 3D–3D comparison of frontal and sphenoid sinuses; in the former case, matches and mismatches yielded RMS values of 0.35 ± 0.23 mm and 2.59 ± 1.79 mm, whereas in the latter case, they amounted up to 0.22 ± 0.11 mm and 2.16 ± 0.57 mm, respectively [13,21]. In the present study, the same values for matches and mismatches were 0.25 ± 0.18 mm and 2.32 ± 0.81 mm, respectively. This result may suggest that the analyzed paranasal sinuses have a similar individualizing potential which makes them an ideal structure for AM–PM comparison in the context of personal identification and in forensic application as evidence in court. In addition, as in other studies applying the same methodology to frontal and sphenoid sinuses, the role of possible differences in the size of paranasal sinuses according to sex was excluded, as match and mismatch comparisons were obtained separately from the two sexes. This choice was required as maxillary sinuses are usually larger in males than in females [29,30,31,32,33,34], and the comparisons in a mixed sample may lead to higher values of RMS in cases of mismatches. Anyway, the statistical analyses did not show any significant difference in RMS values in males or females. So far, reports have analyzed the potential of single paranasal sinuses in personal identification (making comparisons of frontal, sphenoid and maxillary sinuses separately); as for the simultaneous use of right and left sinuses, this approach was applied to frontal and sphenoid sinuses [13,14,21]. No study has verified yet if using more structures for 3D–3D superimposition will increase the reliability of identification. Arguably, we expect that increasing the number of points included in models to compare would lead to a higher difference in the RMS value between matches and mismatches. The abovementioned hypothesis may be valid also, considering both right and left maxillary sinuses. However, this will be confirmed by further studies. 

The present study presents some limitations, nonetheless. AM and PM groups are fictitious, as the 3D models of maxillary sinuses included derive from the same CT scan. This is due to the limited number of subjects who undergo CT scans twice, which may limit the sample size. This is a crucial drawback, as the maxillary sinus may be affected by modifications in shape and size with time. In fact, although the size of the maxillary sinus tends to stabilize after the second decade of life, in adulthood, the volume tends to decrease, probably because of a loss of minerals in the bone matrix with the consequent collapse of bone structures surrounding the air cavity [30]. In addition, the shape and size of the maxillary sinus may vary especially because of teeth loss; in fact, partially and completely edentulous patients show larger maxillary sinuses than dentate subjects, especially in older age groups [31]. Moreover, the extraction of posterior maxillary dental elements proved to increase the size of the maxillary sinus, especially if roots of teeth protrude into the air cavity [32]. In both cases, the expansion of the maxillary sinus may be justified by the lack of mechanical stimulation of alveolar bone by dental elements with its consequent resorption [33]. Moreover, the maxillary sinus is frequently affected by sinusitis, with consequent opacification of the air space, although it does not necessarily modify the sinus shape; therefore, the presence of sinusitis often requires a differential segmentation of the air space through a semi-automatic approach and the opacified volume through a manual approach [45] with obvious complications in extracting a unique and homogeneous model. Future studies need to assess the surface modifications of maxillary sinuses with time and in edentulous subjects. The study sample was selected from a hospital database in Northern Italy, arguably, the ancestry of the individuals is White. Since the literature has demonstrated variation in morphometric features among human populations [22,23,24,25,26], further research may investigate whether inter-population variability, as well as variation in sinus size [46], may affect the identification performance of the method. Additional limitations are related to the acquisition means employed to isolate the 3D models. It can occur that images of maxillary sinuses differ between AM and PM material, because they were produced with different technologies, such as CT and an MRI scan. Although accurate virtual models of anatomical structures can be produced with different techniques [46,47,48], potential variability of the source material and its effect on the segmentation, the final 3D volumes and, eventually, the use of maxillary sinuses for personal identification of unknown bodies and human remains, may be explored in the future. 

## 5. Conclusions

By providing novel data and using up-to-date technologies, this paper demonstrated the reliability of maxillary sinuses in personal identification. The results showed that RMS distance values of maxillary sinuses from the same individual are significantly lower than those of different subjects, pointing out their individualizing properties. Thus, this study contributed to the expanding research field that consider 3D models of anatomical landmarks as reliable structures that may be included in the well-established procedure of identification of the deceased. Future studies will verify the practical applicability of maxillary sinuses and 3D–3D superimposition to the forensic practice. 

## Figures and Tables

**Table 1:** RMS (root mean square), SD (standard deviation), maximum and minimum values of point-to-point distance between 3D models belonging to the same subject (matches) and different subjects (mismatches) according to sex and side: all measurements are expressed in mm.

mm		Males		Females	
		Matches	Mismatches	Matches	Mismatches
Right side	RMS	0.24	2.45	0.21	2.22
	SD	0.16	0.91	0.14	0.73
	Max	0.89	5.24	0.87	3.93
	Min	0.07	1.29	0.03	1.18
Left side	RMS	0.29	2.43	0.27	2.19
	SD	0.21	0.82	0.20	0.74
	Max	0.93	5.45	0.79	4.40
	Min	0.04	1.15	0.02	1.12

**Table 2:** Results of three-way ANOVA test applied to RMS values for the assessment of differences according to sex, side or group (matches–mismatches) together with possible interactions: *: statistically significant differences (*p* < 0.017).

	F	*p*	Eta-Squared
Sex	4.839	0.028	0.012
Side	0.076	0.783	0.000
Group (matches–mismatches)	1262.852	<0.001 *	0.763
Sex × side	0.000	0.984	0.000
Sex × group	3.391	0.066	0.009
Side × group	0.370	0.543	0.001
Sex × side × group	0.002	0.962	0.000

## Data Availability

Data are available upon request.
